# Editorial: Understanding the key border: Structure, function, and dynamics of the plant nuclear envelope

**DOI:** 10.3389/fpls.2022.998823

**Published:** 2022-08-03

**Authors:** David E. Evans, Katja Graumann

**Affiliations:** Department of Biological and Medical Sciences, Faculty of Health and Life Sciences, Oxford Brookes University, Oxford, United Kingdom

**Keywords:** nuclear envelope, nuclear pore complex, plant lamina, LINC complex, nuclear dynamics, chromosome dynamics

The Nuclear Envelope is a hallmark of eukaryotic cells. Despite its emerging role as a key structural and signaling platform, the plant NE remains one of the least understood membrane systems. This Frontiers Research Topic aims to highlight recent advances made to examine the role of the nuclear envelope (NE) as the “key border” in plants. Exploring this border is now proving hugely rewarding, with implications for many aspects of cell function and plant responses to the environment. Better understanding of this membrane system involves understanding its physical connections and its signaling and transport functions. Together they underpin its many functions- as an anchor and shaper; as a transport hub; and as a signaling center. The papers in this Topic provide current knowledge on all these aspects and a valuable basis for further advances.

Three reviews provide insights into processes and structures regulating the dynamics of the plant nucleus. Goto et al. consider factors affecting the shape and size of nuclei with a focus on the proteins of the nuclear membrane and their interactors. Bishop et al. review NE involvement in positioning chromosome domains, in development and in response to biotic and abiotic factors and associated transcriptional patterns. Ludke et al. bring together the proteins discussed in other papers in the Topic with nuclear pore complex (NPC) function, discussing the transport independent functions of NPCs and NE-associated proteins in gene expression and chromatin organization. Together these reviews give background on key components of the NE as well as highlighting future directions for research.

Five original papers address specific proteins at the NE. These are members of the Gamma Tubulin Interacting Family (GIPs); members of the Linker of Nucleoskeleton and Cytoskeleton (LINC complex); and members of the CRWN (Crowded Nucleus) family. GIPs, located in the inner and outer nuclear membrane (INM and ONM) are essential for the recruitment of γ-tubulin complexes at microtubule organizing centers, in the mitotic spindle and for the architecture of centromeres. LINC complexes bridge the NE, with ONM localized Klarsicht/ANC-1/Syne homology (KASH) domain proteins interacting with INM localized SUN domain proteins. These in turn link to the nucleoskeleton and chromatin at the INM whereas KASH proteins connect the cytoskeleton at the ONM. CRWN family proteins form part of the plant nucleoskeleton with attachment to the LINC complex and to NPC components.

Singh et al. present evidence for GIP1 and GIP2 in anchoring chromosome regions to the nuclear periphery. Using DNA damage response as their model, *gip1gip2* double mutants were shown to have aberrant nuclear shape and constitutive DNA damage, indicated by the presence of histone variant γ-H2AX, found at DNA double strand breaks and involved in recruiting DNA repair machinery. Rescue of the double mutant resulted in co-location of γ-H2AX with RAD51at the nuclear periphery. Taken together, the results suggest a role for GIPs in DNA replication and DNA repair and associated chromatin organization.

Three papers address the role of the NE in guard cell response to environmental conditions with large-scale rapid reorganization of the cytoskeleton. Biel, Moser, Meier explore the role of Arabidopsis SUN-interacting nuclear envelope proteins 1 and 2 (SINE1 and SINE2) and their interaction with the cytoskeleton in stomatal guard cells. SINE1 and SINE2 are KASH domain proteins providing efficient NE anchors. *SINE1* and *SINE2* mutants have previously been shown to have an aberrant response to abscisic acid (ABA) attributed to Ca^2+^ and F-actin. Biel, Moser, Meier describe a functional relationship with the microtubule cytoskeleton, showing that SINE1 and SINE2 are required for MT reorganization. In a second paper, Biel, Moser, Groove et al. provide further evidence for the role of SINE1 and SINE2 during ABA-induced stomatal closure, this time exploring the actin cytoskeleton. They show a number of effects of *SINE1* and *SINE2* mutants which strongly suggest roles at an early stage in the ABA induced closure response. McKenna et al., studied proteins known to be associated with the LINC complex, but in the monocot *Zea mays*. Of the three proteins studied, two were homologs of members of the CRWN family (NCH1 and NCH2) and one (MKAKU41) a homolog of the Arabidopsis CRWN interactor KAKU4. Localization to the nuclear periphery was shown using transient, heterologous expression and MKAKU41 shown to interact with LINC component Arabidopsis AtSUN2. NE structure was disrupted by altered expression of the three proteins with invagination and deformation of the nuclear membrane. Severing the nucleoskeleton to LINC connection resulted in reduced severity of NE disruption, while severing the cytoskeleton to LINC connection resulted in increased severity.

Wang et al. selected the liverwort *Marchantia polymorpha* as their experimental system as it has a single CRWN homolog, MpNMCP, This was shown to be localized to the nuclear periphery. While a loss of function mutant of MpNMCP shows reduced growth rates and an aberrant thallus shape, it does not show the altered nuclear morphology of mutants of some CRWN family members in other species. Transcriptomics suggests a role in response to biotic and abiotic stress. An indirect effect, seen in MpNMCP mutants, was dislocation of the male sex chromosome, which is decondensed, rather than being tethered to the nuclear periphery.

This Frontiers Research Topic provides a valuable picture of the NE as a “key border”. Future work must continue to add to the dynamic, physical and mechanistic connections of GIPs, LINC components, NPCs and components of the nucleoskeleton and chromatin. It becomes clear that this sophisticated multi-functional border holds a key role in many important plant responses- from regulation of gene expression to response to biotic and abiotic stress and one that will feature increasingly in future research.

## Author contributions

All authors listed have made a substantial, direct, and intellectual contribution to the work and approved it for publication.

## Conflict of interest

The authors declare that the research was conducted in the absence of any commercial or financial relationships that could be construed as a potential conflict of interest.

## Publisher's note

All claims expressed in this article are solely those of the authors and do not necessarily represent those of their affiliated organizations, or those of the publisher, the editors and the reviewers. Any product that may be evaluated in this article, or claim that may be made by its manufacturer, is not guaranteed or endorsed by the publisher.

